# Strategies for Enhancing and Preserving Anti-leukemia Effects Without Aggravating Graft-Versus-Host Disease

**DOI:** 10.3389/fimmu.2018.03041

**Published:** 2018-12-21

**Authors:** Ying-Jun Chang, Xiang-Yu Zhao, Xiao-Jun Huang

**Affiliations:** ^1^Peking University People's Hospital & Peking University Institute of Hematology, Beijing Key Laboratory of Hematopoietic Stem Cell Transplantation, Beijing, China; ^2^Peking-Tsinghua Center for Life Sciences, Beijing, China

**Keywords:** allogeneic stem cell transplantation, graft-versus-leukemia, graft-versus-host disease, relapse, G-CSF

## Abstract

Allogeneic stem cell transplantation (allo-SCT) is a curable method for the treatment of hematological malignancies. In the past two decades, the establishment of haploidentical transplant modalities make “everyone has a donor” become a reality. However, graft-versus-host disease (GVHD) and relapse remain the major two causes of death either in the human leukocyte antigen (HLA)-matched transplant or haploidentical transplant settings, both of which restrict the improvement of transplant outcomes. Preclinical mice model showed that both donor-derived T cells and natural killer (NK) cells play important role in the pathogenesis of GVHD and the effects of graft-versus-leukemia (GVL). Hence, understanding the immune mechanisms of GVHD and GVL would provide potential strategies for the control of leukemia relapse without aggravating GVHD. The purpose of the current review is to summarize the biology of GVHD and GVL responses in preclinical models and to discuss potential novel therapeutic strategies to reduce the relapse rate after allo-SCT. We will also review the approaches, including optimal donor selection and, conditioning regimens, donor lymphocyte infusion, BCR/ABL-specific CTL, and chimeric antigen receptor-modified T cells, which have been successfully used in the clinic to enhance and preserve anti-leukemia activity, especially GVL effects, without aggravating GVHD or alleviate GVHD.

## Introduction

Allogeneic hematopoietic stem cell transplantation (Allo-HSCT) remains a potentially curative therapeutic strategy for hematological malignancies (1–4). Currently, for patients who require transplantation, but have no related or unrelated donors with matching human-leukocyte antigen (HLA), haploidentical HSCT is an alternative modality, allowing everyone to have a donor (5, 6). Allo-HSCT benefits these malignancies due to a graft-versus-leukemia (GVL) effect that is mainly mediated by donor-derived alloreactive T cells and/or natural killer (NK) cells (7–12). However, T cells are also responsible for acute and/or chronic graft versus-host disease (GVHD), which leads to significant morbidity and mortality (13–15). Although the depletion of T cells from allografts alleviates GVHD either in human leukocyte antigen (HLA)-matched transplant settings or in HLA-haploidentical transplant modalities, removal of these cells results in increased graft failure and increased rates of leukemia relapse (16). Unfortunately, the immunosuppressive agents used for the prophylaxis and treatment of GVHD can also reduce the beneficial GVL effects. Therefore, the separation of GVL effects from GVHD remains the “holy grail” of allo-HSCT (17–20), which is urgently needed to allow a more effective therapy for hematological malignancies.

The challenge for the separation of GVL effects from GVHD is attributed to the underlying similarity of the alloreactive T responses between the two processes (4, 21, 22). In the past 20 years, great efforts have been made by researchers to elucidate specific distinguishing immune mechanisms of GVL vs. GVHD (23–25). In addition, a number of preclinical experiments have been performed to identify approaches that could be successfully used to separate GVL effects from GVHD (26–41). Clinically, a series of strategies, including donor selection, allograft engineering, adoptive immune cell infusion, and pharmacological agents have been established (42–44). More recently, the use of chimeric antigen receptor T (CAR-T) cells that target tumor cells with a limited capacity for GVHD induction, have been identified for enhancing GVL effects without aggravating GVHD (45–47). Previously, several reviews have been published related to the separation of GVL from GVHD (48–51) The present review briefly summarizes the underlying mechanisms related to GVHD and GVL effects, mainly focusing on recent advances in strategies for enhancing and preserving anti-leukemia activity without aggravating GVHD, especially approaches aimed at the separation of GVL effects from GVHD in preclinical mouse models and in the clinic.

## Mechanisms Relevant to GVHD and GVL Effects

The pathophysiology of acute GVHD had been reviewed by several researchers (13–15), beginning with the activation of host antigen-presenting cells (APCs) by damage-associated molecular patterns and/or pathogen-associated molecular patterns expressed on damaged tissues. Activated host APCs then present host antigens to donor T cells, leading to alloactivation and inflammatory cytokine release, for example Interferon-γ (IFN-γ) and lipopolysaccharides (LPS). These inflammatory cytokines then recruit and induce the proliferation of additional immune effector cells, including Th1, Th2, Th17, neutrophils, and macrophages, which cause tissue injury and inflammation in a reaction that overwhelms any tolerance-promoting response from immune suppressor cells, such as regulatory T cells (Treg) (52), regulatory B cells (Breg) (22), mesenchymal stem cells (MSC) (25), and myeloid-derived suppressor cells (MDSC) (53).

**Figure 1:**
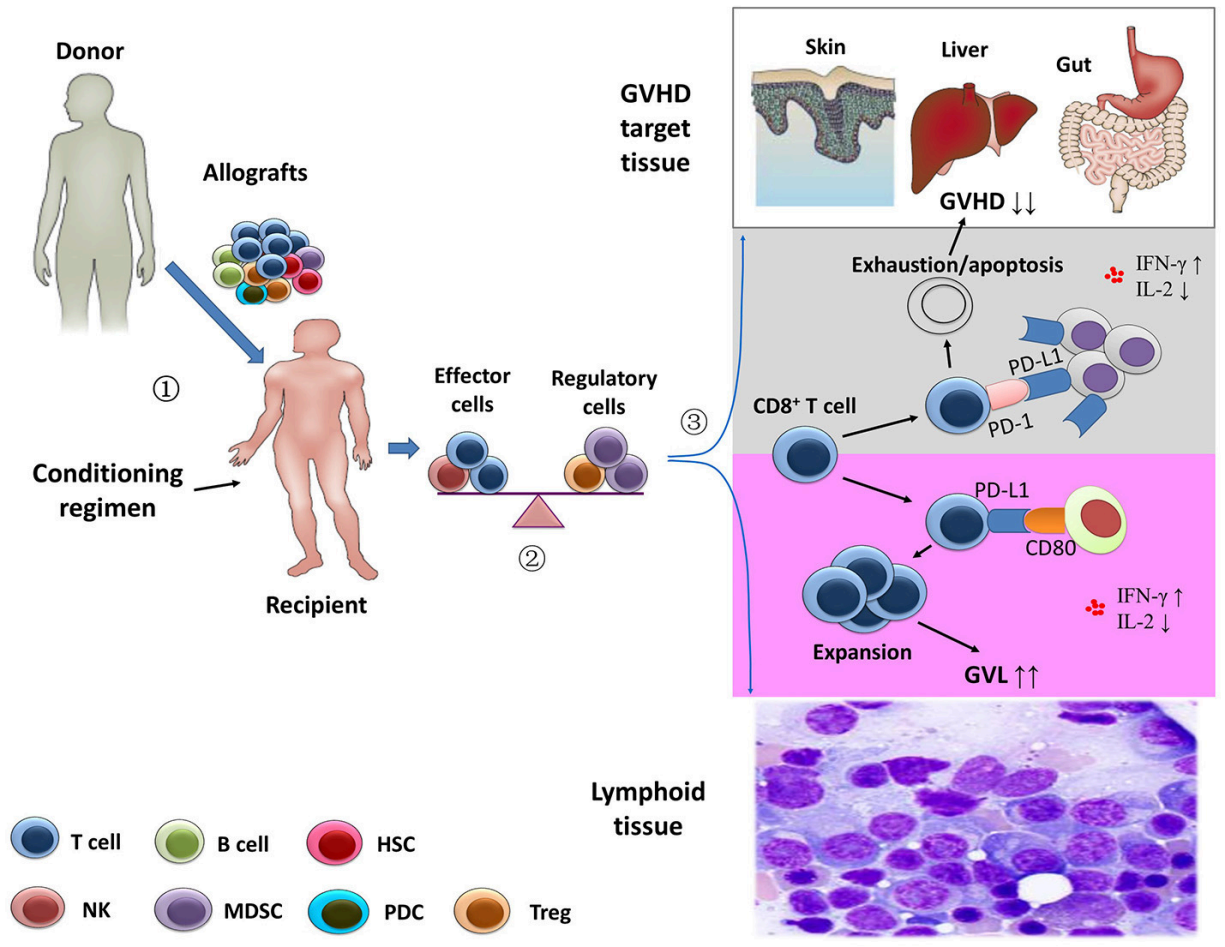
Separation of the graft-versus-leukemia effects from graft-versus-host disease. 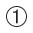 Stem cell harvests obtained from healthy donor were infused in the recipients after the conditioning regimen. 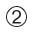 After transplantation, the balance between effector immune cells and regulatory immune cells might contribute to the prevention of graft-versus-host disease (GVHD) and the anti-leukemia activity. 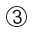 Using animal models, Ni et al. (58) showed that the depletion of CD4^+^ T cells following allogeneic stem cell transplantation significantly increased systemic levels of interferon-γ and decreased interleukin-2. In GVHD-targeted tissues, CD4^+^ T cell depletion enhanced the interaction of PD-L1/PD-1 interactions between CD8^+^ T cells and cells of GVHD-targeted tissues, leading to exhaustion and apoptosis of host-attacking CD8^+^ T cells (gray area). However, the profiles of the cytokines might promote expansion of CD8^+^ T cells via PD-L1/CD80 interactions in lymphoid tissue, resulting in an enhanced anti-leukemia capacity (purple area) (21).

**Figure 2:**
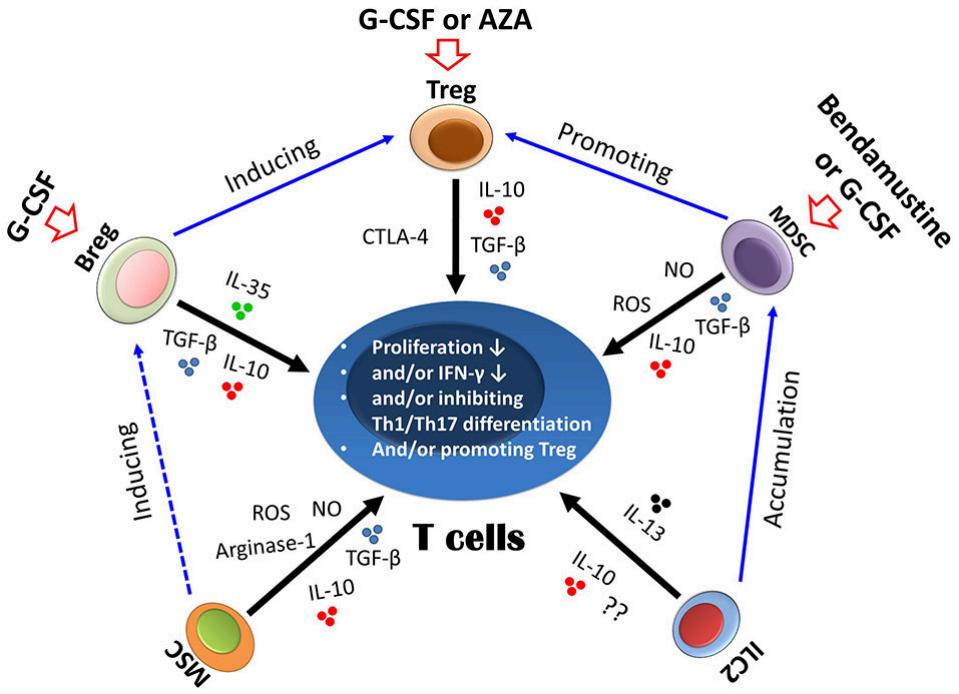
Suppressive mechanism of regulatory immune cells on T cells. Different regulatory cells could suppress T cells either via cytokines, such as IL-10 and TGF-β, or via other molecules, such as arginase-1 and reactive oxygen species (ROS) (indicated by black arrows). The biological interactions between different regulatory cells are indicated by blue arrows. Regulatory immune cells could also be induced by a number of approaches, such as granulocyte colony-stimulating factor (G-CSF), azacitidine (AZA), and bendamustine (indicated by red arrows). Treg, regulatory T cells; Bregs, regulatory B cells; MSCs, mesenchymal stem cells; MDSCs, myeloid-derived suppressor cells; ILC2, group 2 innate lymphoid cells.

**Figure 3:**
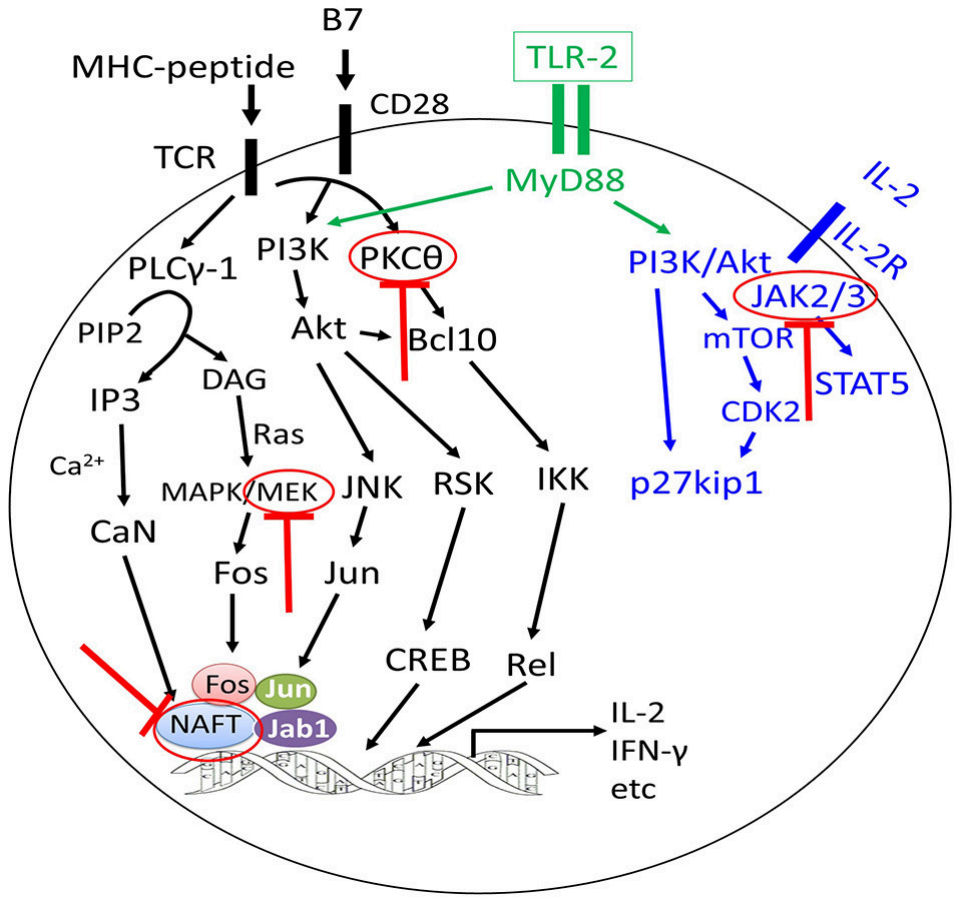
Approaches to separate GVL effects from GVHD using inhibitors targeting different signaling pathways of T cells. The graft-versus-leukemia effects could be enhanced or preserved by targeting different signaling pathways of T cells without aggravating graft-versus-host disease (GVHD) or with alleviation of GVHD (highlighted by red colors).

The mechanisms underlying GVL are of interest (8, 54), as both T cells, natural killer (NK) cells, and cytokines, such as IFN-γ and tumor necrosis factor-α, possess anti-leukemia activity. Two molecular path ways, including perforin and Fas, are mainly used by T cell to mediate cytotoxicity. CD3^+^CD4^+^ T cells utilize the Fas pathway and CD3^+^CD8^+^ T cells use both, while NK cells employ the perforin pathway. Interestingly, all of these cells also play an antileukemia role via cytokine release. Recently, more attention has been focused on the role of γδT cells (55), and iNKT cells in GVL effects. The target antigens for alloreactive T cells include major histocompatibility complex (MHC) and multiplex immunohistochemistry (miHC), or leukemia-associated antigens (56). The importance of MHC and miHC antigens in GVL is underlined by the close association between GVHD and GVL, although selective miHC antigens are considered to be attractive targets for anti-leukemia immunotherapy. More recently, a number of studies have demonstrated that the overall balance between regulatory cells, including Treg, MDSC, and effector cells might be related to the extent of organ damage in GVHD settings and the effects of GVL in anti-leukemia settings (Figures 1–**3**) (21, 22, 25, 52, 53, 57).

## Strategies For Enhancing and Preserving Anti-Leukemia Effects Without Aggravating GVHD in Preclinical Models

Several strategies, such as the use of cytokines (59), selectively depletion of alloreactive T cells, regulatory immune cells (60, 61), and pharmacological agents, such as bortezomib and azacitidine (AZA), have been investigated to enhance and preserve the anti-leukemia effects without aggravating GVHD after allo-HSCT (Table 1) (31, 72, 73).

**Table 1:** Representative approaches for the separation of GVHD and GVL in preclinical models.

**Strategies**	**Authors,yr**	**Approaches**	**Mechanisms**	**References**
Cytokines	Teshima et al., 1999	Interleukin-11	IL-11 selectively inhibited CD4-mediated GVHD, while retaining both CD4- and CD8-mediated GVL.	(41)
	Couturier et al., 2013	IL-22	The absence of T-cell-derived IL-22 led to a reduction of inflammatory CD8 T cells and an expansion of Treg cells in lymphoid organs as well as a reduction of inflammatory mediators both systemically and in aGVHD target organs, both of which resulted in decreased aGVHD severity without compromising GVL effects.	(59)
	Liu et al., 2015	IL-35	IL-35 expression leads to the Treg expansion and suppression of Th1 cytokine production, which alleviates aGVHD and retains GVL effects.	(62)
	Banovic et al., 2009	Multipeg-G-CSF	Multipeg–G-CSF could modulate immune function, characterized by the generation of regulatory myelogenous and T cell populations and Th2 differentiation, as well as improve GVL via activation of invariant natural killer (iNK) T cells and enhancement of CTL function.	(24)
	Morris et al., 2005	Potent G-CSF analogs	Mobilization with potent G-CSF analogs thus allowed concurrent enhancement of NKT cell numbers and activities, promoting host DC activation and subsequent CD8-dependent GVL effects while promoting the generation of regulatory T cells to prevent CD4-dependent GVHD.	(19)
Depletion of alloreactive cells	Zheng et al., 2008	Naïve CD4^+^ T cells	TEMs did not induce high systemic levels of TNF-α and IFN-γ in recipients, as did TNs. In ddition, a greater fraction of TNs produced IFN-γ. GVL mediated by CD4+ TNs was intact even when both perforin- and FasL-mediated killing were prevented.	(63, 64)
Adoptive transfer of immune cells	Ghosh et al., 2017	CAR-T cells	Allogeneic donor CD19-specific CD28z CAR T cells promote anti-lymphoma activity, with minimal GVHD.	(40)
	Song et al., 2018	NK cells	IL-12/15/18-preactivated NK cells predominantly mediated the lysis of donor allo-reactive T cells to inhibit aGVHD without promising GVL effects.	(20)
Regulatory immune cells	Sato et al., 2003	Regulatory DCs	Allogeneic regulatory DC regulation of the cytotoxic activity of transplanted CD8^+^ T cells, which failed to cause acute GVHD, might be sufficient to cause an efficient GVL effect.	(61)
	Heinrichs et al., 2016	Tregs	Harnessing the unique differences between alloreactive CD4^+^ and CD8^+^ iTregs could create an optimal iTreg therapy for GVHD prevention with maintained GVL responses.	(57)
	Li et al., 2014	MSCs	Directing the migration of MSCs by CCR7 from their broad battle field (inflammatory organs) to the modulatory center (SLOs) of immune response could attenuate GvHD while preserving the GvL effect.	(25, 27)
	Highfill et al., 2010	MDSCs	MDSCs generated in the presence of IL-13 could inhibit GVHD, migrate to sites of allopriming, and limit the activation and proliferation of donor T cells, but they did not diminish the GVL effect of donor T cells.	(53)
	Darlak et al., 2013	pDCs	Enrichment of pDCs might augment GVL without increasing GVHD is through the production of IFN-α and/or IL-13 by pDCs.	(60)
Signaling pathway	Vaeth et al., 2015	Nuclear factor of activated T cells	Ablation of NFAT1, NFAT2, or a combination of both resulted in ameliorated GVHD due to reduced proliferation, target tissue homing, and impaired effector function of allogenic donor T cells. In addition, the beneficial antitumor activities were largely preserved in NFAT-deficient effector T cells.	(65)
	Haarberg et al., 2013	Inhibition of PKCα and PKCθ	Inhibition of PKCα and PKCθ impaired donor T-cell proliferation, migration, and chemokine/cytokine production and significantly decreased GVHD, but spared T-cell cytotoxic function and GVL effects.	(66)
	Schutt et al., 2018	Inhibition of the IRE-1α/XBP-1 pathway	Inhibition of the IRE-1a/XBP-1 pathway regulated B-cell activation and function and prevented the development of cGVHD while preserving GVL.	(67)
	Itamura et al., 2016	RAS/MEK/ERK pathway	MEK inhibitors affected human T cells in a memory stage-dependent manner, i.e., they selectively inhibited naive and central memory T cells while sparing effector memory T cells.	(68)
Pharmacological agents	Sun et al., 2004	Proteasome inhibitor	Bortezomib might rapidly induce the preferential deletion of the very high-affinity alloreactive T cells, thus allowing expansion of the remaining T cells that maintain GVT responses yet have a reduced potential for promoting GVHD.	(38)
	Strokes et al., 2016	Bendamustine	BEN alleviated GVHD via enhancing MDSC suppressive function without promising GVL effects.	(69)
	Choi et al., 2010	Azacitidine	AzaC could mitigate GVHD while preserving GVL by peripheral conversion of alloreactive effector T cells into FOXP3^+^ Tregs and epigenetic modulation of genes downstream of Foxp3 required for the suppressor function of Tregs.	(70)
	Ehx et al., 2017	Azacitidine	AZA significantly decreased human T-cell proliferation as well as IFN-γ and TNF-α serum levels, and it reduced the expression of GRANZYME B and PERFORIN 1 by cytotoxic T cells, leading to prevention of GVHD without compromising GVL effects.	(71)
Others	Ghosh et al., 2013	Promyelocytic leukemia zinc finger	PLZF-TG T cells mediated less GVHD due to Fas-mediated fratricidal regulation and the biphenotypic TH1/TH2 response leading to limited alloreactive expansion, and an intact GVT activity.	(72)
	Marcondes et al., 2014	a-1-antitrypsin	Treatment of transplant donors with human AAT resulted in an increase in IL-10 messenger RNA and CD8^+^CD11c^+^CD205^+^MHC II^+^DCs, and the prevention or attenuation of acute GVHD in the recipients. The GVL effect was maintained or even enhanced with AAT treatment of the donor, mediated by an expanded population of NK1.1^+^, CD49B^+^, CD122^+^, and CD335^+^ NKG2D-expressing NK cells.	(35)
	Wu et al., 2015	MicroRNA-17-92	Blockade of miR-17 or miR-19b in this cluster significantly inhibited alloreactive T-cell expansion and IFN-γ production, and it prolonged survival in recipients afflicted with GVHD while preserving the GVL effect.	(73)

GVL, graft-versus-leukemia; GVHD, graft-versus-host disease; Tregs, regulatory T cells; aGVHD, acute GVHD; G-CSF, granulocyte colony-stimulating factor; DCs, dendritic cells; TEM, effector memory T cell; TN, naïve T cells; IFN-γ, interferon-γ; CAR-T, chimeric antigen receptor T; MSCs, mesenchymal stem cells; MDSCs, myeloid-derived suppressive cells; pDCs, plasmoid dendritic cells; TNF-α, tumor necrosis factor-α

### Cytokines

Granulocyte colony-stimulating factor (G-CSF) is widely used during transplantation to mobilize hemopoietic stem cells, which is also a mediator of T cell tolerance (74, 75). Using a murine leukemia model, several researchers have demonstrated that G-CSF mobilization of peripheral blood stem cell transplantation could maintain GVL effects through T cells via a perforin-dependent pathway and/or NKTs and prevent GVHD by reducing systemic levels of LPS and TNF-α as well as inducing a type 2 cytokine profile, CD34^+^ monocyte, and tolerogenic APCs (19, 76). Subsequent studies have shown that allografts mobilized by G-CSF analogs, such as pegylated G-CSF and progenipoietins (engineered chimeric G-CSF and Flt-3L protein), have marked tolerogenic properties that reside in the T cell and APC compartments. Additionally, mobilization with G-CSF analogs allows the concurrent enhancement of NKT cell numbers and activities, promoting host DC activation and subsequent CD8-dependent GVL effects while promoting the generation of Tregs to prevent CD4-dependent GVHD.

Except for G-CSF and its anlogs, other cytokines (77), including KGF, IL-11, IL-18 (28), IL-35 (62), and interleukin-12/23p40 (78), can also be used to separate GVL effects from GVHD in animal models. Moreover, the roles in GVHD of IL-21 and IL-22, two proinflammatory cytokines produced by Th17 cells, have been assessed in several studies (79–81). Couturier et al. (59) and Hanash et al. (79), respectively, demonstrated that IL-22 deficiency in donor T cells and abrogation of donor T-cell IL-21 signaling, could alleviate murine acute GVHD mortality while sparing the GVL effects. Hartung et al. (82) indicated that allografts mobilized by G-CSF plus stem cell factor exerted significantly enhanced antileukemic activity compared with those harvested after treatment with G-CSF alone, suggesting that a combination of different cytokines may be a better strategy for the separation of GVL effects from GVHD.

### Depletion of Alloreactive Cells

To investigate the subsets of T cells that were effector cells with anti-leukemia effects without causing GVHD, a murine transplant model of chronic phase chronic myelogenous leukemia was generated. Zheng et al. (63) found that CD4^+^CD62L^−^CD44^+^CD25^−^ effector memory T cells (CD4^+^ TEMs), but not naïve T cells (T_N_), unprimed to recipient cells mediated GVL without causing GVHD, because they retained key cytolytic functions but lacked other features that are pivotal for initiating GVHD. In another study, Chen et al. (83) reported that sorted CD45RB^+^CD62L^+^CD44^+^ central memory T cells (T_CM_, a mix of CD4^+^ and CD8^+^ cells) did not cause GVHD in a fully MHC-mismatched transplant mouse model. However, using the same model as Chen et al. (83), Zheng et al. (64) demonstrated that highly purified CD8^+^ TCM induced GVHD, albeit less severe than that induced by T_N_. However, CD8^+^ TCM also contribute to GVL.

More recently, using multiple GVHD models (two murine allogeneic HCT models and a human → mouse xenogeneic HCT model), Ni et al. (58) showed that CD4^+^ T cell depletion increased serum IFN-γ levels, leading to an upregulation of PD-L1 in recipient tissues and donor CD8^+^ T cells. In GVHD target tissues, they also found that increased PD-L1/PD-1 interactions between recipient tissues and donor CD8^+^ T cells led to T cell exhaustion and apoptosis, thereby preventing GVHD. In lymphoid tissues, enhanced PD-L1/CD80 interactions between CD8^+^ T cells augmented T cell survival and expansion and preserved the GVL response. In summary, the data reported by Ni et al. (58) suggested that the separation of GVL effects from GVHD could be ascribed to the PD-L1–mediated effect on CD8^+^ T cells depending on whether CD4^+^ T cells were present, the nature of the interacting partner expressed by CD8^+^ T cells, and the tissue microenvironment (Figure 1) (19, 21).

In the clinic, depletion of TN from stem cell allografts has been successfully used to reduce the incidence of chronic GVHD, while preserving the transfer of functional T cell memory (84). Overall, these results suggest that depletion of alloreactive T cells may represent a promising method to preserve GVL effects with decreasing or without causing GVHD.

### Adoptive Transfer of Effective Immune Cells

Adoptive transfer of effective cells represents another strategy for the separation of GVL effects from GVHD. Olson et al. (85) demonstrated that donor T cells exhibited reduced proliferation, CD25 expression, and IFN-γ production in the presence of NK cells. In addition, activated NK cells mediated direct lysis of reisolated GVHD-inducing T cells *in vitro*, both of which lead to the alleviation of GVHD. In addition, the GVL effects were maintained in the presence of NK cells. Using mismatched hematopoietic transplant models, Ruggeri et al. (86) demonstrated, for the first time, that donor-versus-recipient NK cell alloreactivity could eliminate leukemia relapse by killing host lymphohematopoietic cells, and protect patients against GVHD by eliminating recipient-type APCs. The effects of NK cells in enhancing anti-leukemia activity and mitigating GVHD has also confirmed by other researchers. Ghosh et al. (87) reported that adoptively transferred donor-type unsorted TRAIL^+^ T cells could potentially enhance the curative potential of allo-HSCT by increasing the GVT response via fratricide of alloactivated T cells and suppressing GVHD through limiting alloreactive T cell expansion.

Recently, CAR T-cells have been shown to possess a novel adoptive immune therapy (40, 88). Both allogeneic and syngeneic CAR T cells show initial expansion as effector T cells. Jacoby et al. (88) found that, in a mouse model, CAR-mediated acute GVHD was only observed in the presence of leukemia, suggesting that CAR-target interactions induced GVHD. Additionally, Ghosh et al. (40) demonstrated that allogeneic donor CD19-specific CD28z CAR T cells could promote anti-lymphoma activity by non-alloreactive cells, which retained activity against CD19^+^ targets, with minimal GVHD by exhaustion and eventual deletion of the alloreactive CAR-T cells. They also reported that first-generation and 4-1BB-costimulated CARs increased GVHD. Overall, the data obtained from the mouse models suggest that CAR T cells could be used to enhance the anti-leukemia response, although its' effects on GVHD remain controversial.

### Regulatory Immune Cells

Regulatory cell subsets, including Tregs, Bregs, MDSCs, and MSCs, may not only control immune homeostasis, but they also reduce detrimental T cell responses to foreign antigens. In 2003, Edinger et al. (52) observed that, in a mouse model, CD4^+^CD25^+^ Tregs could suppress the early expansion of alloreactive donor T cells, their IL-2-receptor alpha-chain expression and their capacity to induce GVHD without compomising their GVT effects, mediated primarily by the perforin lysis pathway of T conv cells. Interestingly, recipient-type specific Tregs could also control GVHD while favoring immune reconstitution and maintaining GVL effects. (89) In addition, Zheng et al. (90) reported that *ex vivo*-induced CD8^hi^ Tregs controlled GVHD in an allospecific manner by reducing alloreactive T cell proliferation as well as decreasing inflammatory cytokine and chemokine secretion within target organs through a CTLA-4-dependent mechanism in humanized mice. Currently reported data in the literatures suggest that Tregs might be the most important regulatory cells in preventing GVHD (4) through a series of approaches, including aurora A/JAK2 inhibition (91, 92), selective TNFR2 activation (93), DR3 signaling modulation (94), activated protein C signals (95), and IL-2 (96), which can be used to alleviate GVHD through a Tregs-dependent mechanism.

MDSCs are a heterogeneous group of immature immunosuppressive cells of the myeloid lineage, which can induce immunosuppressive cells such as Tregs and skew macrophages toward a proinflammatory type 2 phenotype via IL-10 production. MDSCs can also suppress T-cells via arginase-1, NO, reactive oxygen species, heme oxygenase-1, TFG-β and IL-10, as well as promote Tregs. Highfill et al. (53) found that MDSCs generated in the presence of IL-13 could inhibit GVHD, migrate to sites of allopriming, and limit the activation and proliferation of donor T cells as well as induce a type 2 T cell response that was indispensable for GVHD prevention, but they did not diminish the GVL effect of donor T cells.

Another type of regulatory immune cells is the MSCs, which can inhibit the activation, proliferation, and function of T cells via arginase-1, NO, reactive oxygen species, chemokines, TGF-β, and IL-10. Interestingly, *in vivo* experiment have shown that MSCs are actively induced to undergo perforin-dependent apoptosis by recipient phagocytes that produced indoleamine 2,3-dioxygenase, which was essential to initiate MSC-induced immunosuppression (97). Directing the migration of MSCs by CCR7 from their broad battle field (inflammatory organs) to the modulatory center of the immune response could attenuate GVHD by exerting immunosuppressive effects on T cells, while preserving GVL effects by sparing the NK cell activity that contributes to GVL effects (25, 98).

Bregs can suppress immunopathology by prohibiting the expansion of pathogenic T cells and other pro-inflammatory lymphocytes through the production of IL-10, IL-35, and TGF-β (99). Our group showed that, in the acute GVHD mouse model, cotransplantation of Bregs prevented onset by inhibiting Th1 and Th17 differentiation and expanding regulatory T cells. In the GVL mouse model, Bregs contributed to the suppression of acute GVHD but had no adverse effects on GVL activity (22).

Excluding the abovementioned regulatory cells, group 2 innate lymphoid cells (ILC2) make up a large portion of the ILC population, which can polarize T cells to Th2 cells by secreting IL-4, and macrophages or DCs to an macrophage 2 or type 2 chemokine-secreting phenotype by secreting IL-13, respectively (100). ILC2 can alleviate GVHD by reducing donor Th1 and Th17 cells as well as accumulating MDSCs mediated by IL-13. Moreover, ILC2 do not inhibit the GVL response (101).

In summary, these preclinical studies suggest that cotransplantation or adoptive transfer of regulatory cells could be successfully used to alleviate GVHD without compromising the GVL effects. Therefore, pilot studies are warranted to evaluate the safety and feasibility of these regulatory cells in preventing and/or treating GVHD as well as preserving GVL effects in clinic.

### Signaling Pathways

Several signaling pathways have been demonstrated to be correlated with T cell function. Janus kinases (JAKs) are intracellular signaling components of many type I/II cytokines (102, 103). There are 4 members of the JAK family that regulate the development and function of immune cells, including DCs, macrophages, T cells, B cells, and neutrophils, of which JAK1, JAK2, and JAK3 may be most relevant for the pathophysiology of GVHD (51). In murine models of GVHD and leukemia or lymphoma relapse, treatment with ruxolitinib reduced GVHD in the skin, liver, and gastrointestinal organs while preserving GVL activity, leading to improved survival (44, 104, 105). Betts et al. (91) found that the transfer of JAK2^−−/−−^ donor T cells to allogeneic recipients led to attenuate GVHD by inhibiting Th1 differentiation, promoting Th2 polarization, and increasing and/or stabilizing CD8^+^ iTreg, yet it maintained GVL effects (106). In addition, pacritinib, a multikinase inhibitor with potent activity against JAK2, could significantly reduce GVHD and xenogeneic skin graft rejection in distinct rodent models and maintain donor anti-tumor immunity. Overall, these data suggest that JAK inhibition or other compounds, such as TG101348 (92), represents a new and potentially clinically relevant approach to separate GVL effects from GVHD.

Excluding JAKs, increasing data have demonstrated that targeting signaling pathways, such as the PKCα and PKCθ (66), MEK (68), NFAT (65), and IRE-1a/XBP-1 pathway (67), ikaros (107), toll-like receptor/myeloid differentiation factor 88 (108), DR3 signaling (94), and activated protein C signals (95), might provide strategies for alleviating GVHD, while enhancing or without compromising the GVL effects.

### Pharmacological Agents

The roles played by biological agents in the separation of GVL effects from GVHD have been investigated in animal models (38, 71). Sun et al. (38) demonstrated that bortezomib might rapidly induce the preferential deletion of very high-affinity alloreactive T cells, thus allowing for expansion of the remaining T cells to maintain GVT responses yet with a reduced potential for promoting GVHD. Ehx et al. (71) found that AZA significantly decreased human T-cell proliferation as well as IFN-γ and TNF-α serum levels, and it reduced the expression of GRANZYME B and PERFORIN 1 by cytotoxic T cells, leading to the prevention of GVHD. AZA could also induce the expression of tumor antigens by AML cells, leading to the generation of donor-derived tumor specific cytotoxic T cells, which have been demonstrated to prevent AML relapse (70). In addition, Stokes et al. (69) reported that bendamustine could alleviate GVHD by enhancing MDSC suppressive function without compromising GVL effects.

Caballero-Velázquez et al. (30) showed that the combination of sirolimus and bortezomib synergistically inhibited both the activation and proliferation of stimulated T cells. Additionally, the production of Th1 cytokines (IFN γ, IL-2, and TNF-α) was significantly inhibited. This effect was due, at least in part, to the inhibition of Erk and Akt phosphorylation. *In vivo*, the combination reduced the risk of GVHD without hampering GVL effects, as shown in mice that received GVHD prophylaxis with sirolimus plus bortezomib infused with tumor WEHI cells plus C57BL/6 donor BM and splenocytes. Overall, this study suggests a synergistic effect of the combination different pharmacological agents to prevent GVHD while maintaining the GVL effect.

In summary, experiment results from mouse models suggest that effective and regulatory immune cells play a key role in separation of GVL effects from GVHD. The approaches explored in preclinical settings have demonstrated, for example, that cytokines or inhibitors targeting signaling pathways of T cells might enhance and/or preserve ant-leukemia effects without compromising GVHD through regulating the functions of effective and regulatory immune cells (Figures 2, 3).

## Strategies For Enhancing and Preserving Anti-Leukemia Effects Without Aggravating GVHD in the Clinic

**Figure 4:**
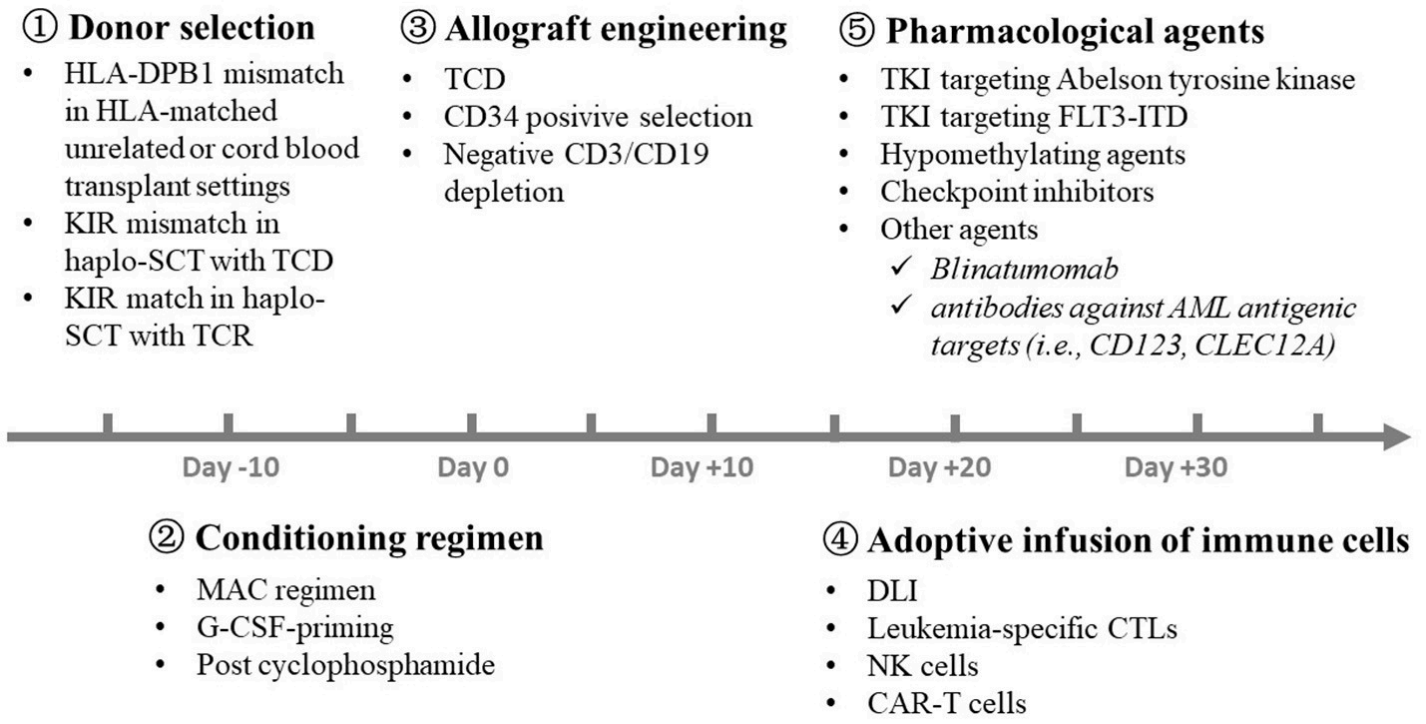
Strategies for the separation of GVL effects from GVHD in the clinic. A number of approaches, including 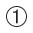
donor selection, 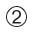
conditioning regimen, 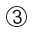
graft engineering, 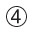
adoptive transfusion of immune cells, and 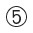 pharmacological agents, have been successfully used in the clinic to separate GVL effects from GVHD at different time point before and after allo-HSCT. GVL, graft-versus-leukemia; GVHD, graft-versus-host disease; HLA, human leukocyte antigen; KIR, killer immunoglobulin-like receptor; SCT, stem cell transplantation; TCD, T cell depletion; TCR, T cell replete; MAC, myeloablative regimen; G-CSF, granulocyte colony-stimulating factor; DLI, donor lymphocyte infusion; NK, natural killer; CAR-T, chimeric antigen receptor T; TKI, Tyrosine kinase inhibitor; AML, acute myeloid leukemia.

Several approaches, including donor selection, conditioning regimens, graft engineering and adoptive transfusion of immune cells, have been successfully used in the clinic to separate GVL effects from GVHD before and after allo-HSCT (Figure 4).

### Donor Selection

In unrelated donor transplantation settings, Kawase et al. (109) suggested that donor selection made in consideration of these results might allow the separation of GVL from acute GVHD in patients with AML, ALL, or those with chronic myeloid leukemia (CML), especially in HLA-DPB1 mismatch combinations. Fleischhauer et al. (110) further demonstrated that avoidance of an unrelated donor with a non-permissive T-cell-epitope mismatch at HLA-DPB1 might contribute to a lower risk of mortality. In cord blood transplant modality, HLA-DPB1 mismatch was also associated with a significant reduction of leukemia relapse (HR 0.61, *P* = 0.001), and no significant effect of HLA-DPB1 mismatch was observed on the risk of acute GVHD, engraftment or mortality (111). Laghmouchi et al. (112) suggested that the allo-HLA-DP-specific T cell repertoire contained T cells with restricted recognition of hematopoietic cells, which might contribute to specific GVL effector reactivity without coincident GVHD (112).

In T cell depleted haplo-SCT settings, Ruggeri et al. (86) showed that increased NK cell alloreactivity in humans, based on the “missing self” model, was associated with a decreased CIR and improved survival in patients with AML but not in patients with ALL. In contrast, Huang et al. (113) following the Beijing Protocol, demonstrated that host MHC class I could determine NK cell responses. The functional recovery of donor-derived NK cells was higher in recipients that expressed ligands for donor inhibitory KIRs, and a high functional NK recovery correlated with better relapse control (114). In haplo-SCT with PT/Cy settings, Shimoni et al. (115) also demonstrated a trend toward higher relapse rates in patients with KIR ligand mismatching (HR 1.36, *P* = 0.09) in a total group of 444 acute leukemia patients. This trend was observed in patients with AML (HR 1.48, *P* = 0.07) but not in those with ALL (HR 0.95, *P* = 0.88).

In summary, these data suggest that donor selection according to HLA-DPB1 mismatch, NK cell alloreactivity, and other variables (116–119), could represent a strategy for the separation of GVL effects and GVHD, although further studies are still needed.

### Conditioning Regimen

More recently retrospective registry studies and some, but not all, prospective randomized trials have demonstrated increased relapse rates in recipients of an RIC compared with an MAC regimen in patients with AML and MDS who underwent allo-HSCT (120, 121). However, these finding remain controversial (3). In a multicenter randomized controlled trial (122), 178 HR-AML patients received haplo-HSCT with conditioning regimens involving recombinant human G-CSF or non-rhG-CSF. The cumulative incidences of acute GVHD, chronic GVHD, transplantation-related toxicity, and infectious complications appeared to be equivalent. The 2-year probabilities of LFS and OS in the G-CSF-priming and non-rhG-CSF-priming groups were 55.1 vs. 32.6% (*P* < 0.01) and 59.6 vs. 34.8% (*P* < 0.01), respectively. This study suggests that the G-CSF-priming conditioning regimen is an acceptable choice for HR-AML patients, which may lead to partially separation of GVL effects from GVHD.

Overall, considering the central importance of regimen in determinng leukemia relapse risk based on the biological characteristics of disease and pretransplantation minimal residual disease (MRD), there remains an urgent need for randomized comparisons of different conditioning regimens to separate GVL from GVHD.

### Allograft Engineering

In a single-arm clinical trial, 35 cases patients with high-risk leukemia received naïve T cell-depleted G-PBSCs following a myeloablative conditioning regimen. GVHD prevention includes tacrolimus immunosuppression alone. Bleakley et al. (42) reported that all the cases engrafted. GVHD in these patients was universally corticosteroid responsive, although the incidence of aGVHD was not reduced. Chronic GVHD was remarkably infrequent (9%) compared with historical rates of ~50% with T cell-replete grafts. Memory T cells in the graft resulted in rapid T cell recovery and the transfer of protective virus-specific immunity. No excessive rates of infection or relapse occurred, and the OS was 78% at 2 years. These results suggest that the depletion of naïve T cells from allografts not only reduces the incidence of cGVHD but also preserves the transfer of functional T cell memory.

To decrease the incidence of GVHD in haploidentical allograft settings, the Perugia group established a protocol that includes TCD and a graft containing a mega-dose of highly purified CD34^+^ cells (average 10 × 10^6^/kg body weight), which is administered following a myeloablative conditioning regimen (86). This protocol ensures a high engraftment rate, despite the HLA barrier, without triggering GVHD. However, the benefit (the absence of GVHD) from this CD34 selected haplotype transplant approach is offset by a very slow immune recovery due to the small number of T cells infused and the ATG application, which result in high rates of opportunistic infections, such as viral and fungal infections, leading to a high TRM. To accelerate immune recovery, the Perugia group demonstrated, for the first time, that the adoptive transfer of Tregs promotes lymphoid reconstitution and improves immunity to opportunistic pathogens without weakening the GVL effects in the TCD haploidentical setting (16). This finding suggests that the adoptive transfer of gene modified T cells and/or pathogen-specific T cells may be needed to improve clinical outcomes. In a phase II study, researchers from Germany found that haplo-SCT with a negative CD3/CD19 depletion and reduced intensity conditioning allowed for a successful transplantation in an older, heavily pretreated patient population (16). The estimated 2-event free survival was 25%. The incidence of grade II-IV aGVHD was 46%, and the incidence of cGVHD was 18%. Therefore, new strategies are needed to further establish novel strategies for the separation of GVL effects and GVHD.

Luznik et al. (123) summarized that *in vivo* cyclophosphamide posttransplantation (PT/Cy) could induce the destruction of peripheral, alloantigen-reactive T cells, while a relative resistance of donor Teff/memory T cells to PT/Cy, as demonstrated in mice, might contribute to the overall reconstitution of peripheral T-cell pools and immune competence over the long term. These results suggest that *in vivo* allograft engineering with PT/Cy represents a novel method for GVL and GVHD separation, and it has been widely used in haploidentical and HLA-matched sibling donor transplant settings (6, 124).

### Adoptive Transfusion of Immune Cells

Currently, adoptive transfusion of immune cells, such as donor lymphocyte infusion (DLI), cytotoxic T lymphocyte (CTLs), NK cells, and CAR-T, had been successfully used to separate GVL effects from GVHD.

#### DLI

In 1990, Kolb et al. (125) first reported sustained remission after DLI in patients with CML who relapsed after allo-HSCT. Since then, DLI had become the mainstay allogeneic cellular therapy. The NCI recommendations list DLI as the routinely considered method for patients who relapsed after allo-HSCT and do not have GVHD (126). Based on immune tolerance induced by G-CSF, such as the ability to polarize T cells from the Th1 to the Th2 phenotype and the hyporesponsiveness of T cells, Huang's group established a modified DLI protocol (127) that includes the following: (i) the use of G-CSF mobilized peripheral blood stem cell harvests (G-PBSCs) instead of a steady lymphocyte infusion; (ii) the introduction of short-term immune suppressive agents, including cyclosporine A (CSA) or methotrexate (MTX), to further decrease the incidence of GVHD. Impressively, the feasibility and efficacy of the modified DLI were confirmed either for treatment or prevention of relapse after haploidentical HSCT (127). Our group also demonstrated that MRD-directed DLI could significantly decreased the relapse rate without aggravating GVHD (128). The use of DLI was also demonstrated in patients who underwent haploidentical HSCT with PT/Cy (129).

Recently, Nikiforow et al. (130) undertook a phase I study of DLI depleted of CD25^+^ T cells in 21 patients with hematologic malignancies who had relapsed after allo-HSCT. Two dose levels were administered: 1 × 10^7^ (*n* = 6) and 3 × 10^7^ CD3^+^ cells/kg (*n* = 15). A median 2.3 log-depletion of Tregs was achieved. Seven subjects (33%) developed clinically significant GVHD by 1 year, including one patient who died. At dose level 1, five subjects had progressive disease and one had stable disease. At dose level 2, nine subjects (60%) achieved or maintained responses (8 CR, 1 PR), including seven with active disease at the time of infusion. A shorter period between relapse and infusion was associated with the response at dose level 2 (*P* = 0.016). The 1-year survival rate was 53% among patients treated with dose level 2. Four of eight subjects with AML remained in remission at 1 year. When compared to unmodified DLI in 14 contemporaneous patients meeting study eligibility, CD25/Treg depletion was associated with a better response rate and improved EFS.

Overall, the available data suggest that DLI represents a widely used approach in prophylactic, pre-emptive therapy and therapy for relapse either in HLA-matched HSCT or in haploidentical transplant settings. Furthermore, CD25/Treg-depleted DLI appears to be feasible and capable of inducing GVL effects without excessive GVHD (131), although multicenter, prospective study are warranted to confirm the results.

#### Leukemia Specific CTLs

Researchers from Italy have investigated the feasibility of expanding/priming p190BCR-ABL–specific T cells *in vitro* by stimulation with DCs pulsed with p190BCR-ABL peptides derived from the BCR-ABL junctional region and alternative splicing, and of adoptively administering them to patients with relapsed disease (132). Three patients were enrolled in this study. Patient 1 was a 61-year-old man experiencing a second molecular recurrence after matched unrelated donor (MUD) alloHSCT and unmanipulated DLIs. Patient 2 was a 30-year-old man diagnosed with Ph^+^ ALLwith hyperleukocytosis and central nervous system (CNS) involvement, experiencing his third hematologic relapse (BM blast 66%, F317L mutation) after MUD-HSCT, DLI, and rescue therapy with nilotinib. Patient 3 was a 62-year-old woman diagnosed with Ph^+^ ALL with CNS involvement, showing persistent molecular disease (last MRD before T-cell therapy 0.1% BCR-ABL/ABL) after induction, maintenance chemotherapy, and prolonged TKI treatment. She was not eligible for alloHSCT due to comorbidities. The results showed no postinfusion toxicity, except for a grade II skin GVHD in the patient who was treated for hematologic relapse. All patients achieved a molecular or hematologic CR after T-cell therapy, upon emergence of p190BCR-ABL-specific T cells in the BM. These results demonstrate that p190BCR-ABL-specific CTLs are capable of controlling treatment-refractory Ph^+^ ALL *in vivo*, and they support the development of adoptive immunotherapeutic approaches with BCR-ABL CTLs in Ph^+^ ALL. Therefore, further studies including large sample sizes are needed to confirm the abovementioned results.

Excuding BCR-ABL CTLs, the anti-leukemia effects of WT1 specific CTL were also observed in 11 relapsed or high-risk leukemia patients who underwent allo-HSCT (133). Chapuis et al. (133) found that CD8^+^ transferred T cells with a memory phenotype could be detected after long-term follow-up. An approach to generate multi-TAA-specific CTLs using peptide libraries of 15-mer peptides overlapping by 11 amino acids spanning the whole amino acid sequence of a target antigen was developed by Weber et al. (134) They also showed that TAAmix-specific CTLs could inhibit the colony formation of leukemia blasts. In summary, leukemia-specific CTLs might be a promising method for enhancing anti-leukemia activity.

#### NK Cells

The role played by NK cells in anti-leukemia activity had been fully investigated in allo-HSCT settings. In a dose-escalation study, Choi et al. (43) showed that, when given 2–3 weeks after haploidentical HSCT, donor-derived NK cells were well-tolerated at a median total dose of 2.0 × 10^8^ cells/kg. In a phase I study, the safety of haploidentical third-party NK cell infusion was further confirmed in 21 patients with high-risk myeloid malignancies who received a preparative regimen with busulfan and fludarabine followed by infusion of IL-2-activated NK cells with a dose ranging from 0.02 to 8.32 × 10^6^/kg. Lee et al. (135) demonstrated that five patients were alive, and 5 and 11 cases had died from transplant-related causes and relapse, respectively. Among the total patients, only 5 cases developed a maximum acute GVHD of grade 2, and 2 cases grade 3 GVHD. These results indicated that the infusion of third-party NK cells was well-tolerated and did not increase the rate of GVHD after allo-HSCT. Ciurea et al. (136) initiated a phase 1 dose-escalation study of membrane-bound interleukin 21-expanded donor NK cells infused before and after haploidentical HSCT for high-risk myeloid malignancies. NK cells were infused on days −2, +7, and +28 posttransplant. All NK expansions achieved the required cell number, and 11 of 13 patients enrolled received all 3 planned NK-cell doses (1 × 10^5^/kg to 1 × 10^8^/kg per dose). No infusional reactions or dose-limiting toxicities occurred. All patients were engrafted with donor cells. Seven patients (54%) developed grade I-II acute GVHD (aGVHD), and no patients developed grade III-IV aGVHD or chronic GVHD. All other patients were alive and in remission at the last follow-up (median, 14.7 months). Overall, this trial demonstrated the production feasibility and safety of infusing high doses of *ex vivo*-expanded NK cells after haploidentical HSCT without adverse effects, increased GVHD, or higher mortality, which was associated with significantly improved NK-cell numbers and function, fewer viral infections, and a low relapse rate posttransplant. Further study deomonstrated that CD56^+^ donor cell infusion after PT/Cy and short-course cyclosporine were feasible with prompt engraftment, rapid reconstitution of CD4^+^ T, Tregs and NK cells and a reduced incidence of relapse and acute GVHD (137).

#### CAR-T Cells

Researchers from Peking University described six ALL patients with no response to modified DLI who received one and two infusions of CAR T cells from haplo-HSCT donors. Five patients (83.33%) achieved MRD-negative remission; one patient was discharged without evaluation after developing severe thrombotic microangiopathies (46, 47, 138). More recently, Anwer et al. (45) performed a systemic review, including 72 patients from seven studies who were treated with donor-derived CAR T cells. The authors reported that the use of donor-derived CAR T cell for relapse prophylaxis, MRD clearance or salvage from relapse is therefore highly effective, and the risk of GVHD flare is very low.

In summary, donor-derived CAR T-cell infusion seems to be an effective and safe alternative method for relapsed B-ALL after haplo-HSCT (47). Therefore, with the definition of multiple antigen targets, such as CD7, CD38. CD138, FLT-3, and B-cell maturation antigen, CAR-T cell could be increasingly used for anti-hematological malignancies.

## Pharmacological Agents

### Tyrosine Kinase Inhibitor (TKI) Targeting Abelson Tyrosine Kinase

Currently, few patients with CML will receive allo-HSCT. Therefore, the use of TKI after transplantation mainly focuses on cases with Ph-positive ALL (139–141). Chen et al. (142) reported that 14 patients who were positive for BCR-ABL1 expression, received imatinib therapy after allo-HSCT. Eight patients became BCR-ABL1-negative at 1 month after imatinib therapy, and only two patients died from hematological relapse. In the nonimatinib-treated group, six of 20 patients relapsed, and five of these patients died from hematological relapse. Here, recommendations for the use of TKIs according to the pre- and post-transplant MRD status by the Acute Leukemia Working Party of the European Society for Blood and Marrow Transplantation are provided as follows (143).

First, for cases with positive pre-MRD, but negative posttransplantation MRD (post-MRD), prophylactic TKI should be administered according to the pretransplantation mutation status, or observation only. If positive post-MRD is detected, imatinib or another TKI can be administered according to the mutation status. If MRD reoccurs within 3 months after transplantation or at a high level, a 2nd generation TKI should be given.

Second, for cases with positive post-MRD not considering the status of pre-MRD, TKI is administered according to mutation status or using 2nd generation TKI.

Third, for cases with both negative pre-MRD and negative post-MRD, prophylactic TKI or observation, if positive post-MRD is detected, imatinib or another TKI can be administered according to the mutation status. If MRD reoccurrs within 3 months after transplantation or at a high level, a 2nd generation TKI should be given.

### TKIs Targeting FLT3-ITD

A number of FLT3 TKIs have been or are being investigated in allo-HSCT settings for FLT3-ITD AML, including sorafenib (144, 145), midostaurin, quizartinib, crenolanib, and gilteritinib (144–149). The mechanism of action of TKIs targeting FLT3 may not only involved in direct tumor cell killing, but also in increased interleukin-15, leadings to an increase in CD8^+^CD107a^+^IFN-γ^+^ T cells with features of longevity (high levels of Bcl-2 and reduced PD-1 levels), which could eradicate leukemia in secondary recipients (146). More recently, Xuan et al. (147) performed a study that enrolled a total of 144 patients with FLT3-ITD AML undergoing allo-HSCT. Depending on whether they were receiving sorafenib before transplantation or sorafenib maintenance after transplantation, patients were divided into 4 groups: patients receiving sorafenib before transplantation (group A; *n* = 36), patients receiving sorafenib after transplantation (group B; *n* = 32), patients receiving sorafenib both before and after transplantation (group C; *n* = 26), and patients receiving sorafenib neither before nor after transplantation (group D; *n* = 50). Xuan et al. (147) showed that the 3-year relapse rates were 22.2, 18.8, 15.8, and 46.1% for groups A, B, C, and D, respectively (*P* = 0.006). The 3-year LFS rates were 69.4, 78.1, 80.4, and 34.8%, respectively (*P* < 0.001). A multivariate analysis revealed that sorafenib before transplantation, sorafenib maintenance after transplantation, and their combined application were protective factors for a lower relapse rate and longer LFS, respectively.

More recent studies have shown that targeting the FLT3-ITD driver mutation with a highly potent and selective FLT3 inhibitor, such as quizartinib, is a promising clinical strategy to help improve clinical outcomes in patients with relapsed or refractory AML (148, 149). Therefore, further studies are needed to investigate the effectiveness of these agents in allo-HSCT settings, especially for the separation of GVL from GVHD.

### Hypomethylating Agents

Hypomethylating agents are used as treatments for relapse and may also be used in pre-emptive interventions after allo-HSCT (150–154). In a phase 1 study enrolling 27 patients with AML post allo-HSCT. Goodyear et al. (155) showed that azacitidine (AZA) both augmented the expansion of regulatory T cells and induced cytotoxic CD8^+^ T-cell responses to several tumor antigens, and leading to hopes that it might facilitate successful cultivation of the GVL response without inducing significant GVHD. In a multicenter retrospective study, Craddock et al. (156) investigated the tolerability and activity of AZA in 181 patients who relapsed after an allograft for AML (*n* = 116) or MDS (*n* = 65). Sixty-nine patients received additional DLI. Forty-six of 157 (25%) assessable patients responded to AZA therapy: 24 (15%) achieved a CR and 22 a PR. In patients who achieved a CR, the 2-year overall survival was 48 vs. 12% for the whole population. The authors suggested that AZA represents an important new therapy in select patients with AML/MDS who relapse after allo-HSCT, thus warranting prospective studies. Moreover, the combination of sorafenib, AZA, and DLI represent a novel direction for the treatment or prevention of relapse without aggravating GVHD after allo-HSCT (151, 157).

Recently, Schroeder et al. (158) retrospectively analyzed data obtained for 36 patients with hematological (*n* = 35) or molecular relapse (*n* = 1) of AML (*n* = 29), or MDS (*n* = 7). Decitabine (DAC) was the first salvage therapy in 16 patients (44%), whereas 20 patients (56%) had previously received 1–5 lines of salvage therapy, including 16 cases who had been treated with AZA. In 22 patients (61%), a median of 2 DLI per patient (range, 1–5) was administered in addition to DAC. As a result, the overall response rate was 25%, including 6 CR (17%) and 3 PR (8%). Three patients within the first-line group achieved CR, while 3 patients receiving DAC as second-line treatment reached CR, including 2 patients with previous AZA failure. The median duration of CR was 10 months (range, 2–33), and none of the patients have relapsed to date. The incidence of acute and chronic GVHD was 19 and 5% (158). These data suggest that DAC may be an alternative to AZA or even a second choice after AZA failure. In summary, hypomethylating agents used alone or in combination with DLI might represent promising approaches for the separation of GVL from GVHD in the clinic.

### Checkpoint Inhibitors

The relapse of hematological malignancies after allo-HSCT can be mediated by high levels of checkpoint receptors, including PD-1 and CTLA-4, on donor derived effective T cells and high expression of cognate ligands on residual leukemia cells (159, 160). In a phase 1 study, Bashey et al. (161). showed that a single dose of ipilimumab (between 0.1 and 3.0 mg/kg) for patients with malignancies who relapsed after allo-HSCT did not seem to cause clinically significant GVHD and achieved responses in 3 patients with lymphoid malignancies. In a subsequent phase 1/2 study, ipilimumab was started at 3 mg/kg but could be escalated to 10 mg/kg (162). Although no objective responses were observed in six patients who received ipilimumab treatment at 3 mg/kg, a total of 13 patients presented a decrease in tumor burden among 22 patients treated at a dose of 10 mg/kg, with four responses persisting for >1 year. Impressively, four patients with extramedullary AML and one patient with smoldering MDS that developed into AML had a complete response. These data suggest a particular sensitivity of AML to ipilimumab treatment after allo-HSCT. Davids et al. (162) also observed that responders showed a reduction of CD4^+^ regulatory T cells with an increase in conventional T cells in peripheral blood as well as an increase in CD62L^−^ effector memory T cells.

A phase 2 investigator-initiated trial enrolled patients with lymphoid malignancies who relapsed after allogeneic HSCT (*n* = 10) and high-risk patients after autologous HSCT (*n* = 7) (163). Both cases received 10 mg of oral lenalidomide daily for 21 days followed by intravenous ipilimumab at 3 mg/kg body weight. The regimen was repeated 4 weeks later for a total of 4 treatments. Khouri et al. (163) demonstrated that 4 of 10 patients in the allogeneic group had complete responses and 3 partial responses. The disease in 6 of 7 patients in the autologous group remains in remission. The authors suggested that the responses might be related to a 2- to 3 -fold increases in inducible ICOS^+^CD4^+^FoxP3^−^ T cells number.

In summary, checkpoint inhibitor used alone or in combination with other methods, such as immunomodulatory agents (163) and CAR-T cells (164), could be promising approaches for the treatment or prevention of relapse after transplantation without aggravating GVHD, although further studies are warranted for confirmation.

### Other Agents

Several other novel agents (165), including histone deacetylase inhibitor (panobinostat), and monoclonal antibodies, such as blinatumomab (a novel bispecific CD19-directed CD3 T-cell engager), as well as antibodies against AML antigenic targets (i.e., CD123, CLEC12A), have been or are currently being investigated for the prevention and treatment of relapse in patients with hematological malignancies who have undergone allo-HSCT. Therefore, further prospective studies are warranted to select optimal methods that are currently available for killing leukemia cells without leading to GVHD.

## Future Directions

In the past two decades, increasing evidence supports the notion that GVL effects could be, at least partially, separated from GVHD both in animal models and in the clinic. Recently, Fanning et al. (18) have demonstrated that Vβ spectratyping can identify T cells involved in antihost and antitumor reactivity and that tumor presensitization can aid in the separation of GVHD and GVL responses. However, no studies have demonstrated the successful use of this technique for separating GVL effects from GVHD in patients who have undergone allo-HSCT. In addition, several other questions remain to be answered in the future. First, although preclinical experiments have demonstrated the feasibility of a number of strategies for enhancing or preserving anti-leukemia activity without compromising GVHD, planned prospective studies are required to evaluate the clinical efficacy and to move these approaches from preclinical research to the standard-of care. Second, it remains uncertain whether the available methods for inducing anti-leukemia activity without causing GVHD can be successfully used in different transplant modalities, especially haploidentical allografts. Third, little is known about the immunological mechanisms underlying the separation of GVL effects from GVHD. Therefore, further studies are imperative.

In summary, with the elucidation of the immune mechanisms of both GVL effects and GVHD, the advances in the establishment of novel approaches for the prevention and/or treatment of leukemia relapse and GVHD, as well as the evaluation of these new methods based on prospective clinical trials, an increasing number of patients will benefit from the successful separation of GVL effects from GVHD, ultimately leading to superior survival.

## Author Contributions

X-JH designed the study. Y-JC and X-YZ collected data and drafted the manuscript. All authors contributed to data interpretation, manuscript preparation, and approval of the final version.

### Conflict of Interest Statement

The authors declare that the research was conducted in the absence of any commercial or financial relationships that could be construed as a potential conflict of interest.
